# Anti-Inflammatory Effects of Compounds from *Cudrania tricuspidata* in HaCaT Human Keratinocytes

**DOI:** 10.3390/ijms22147472

**Published:** 2021-07-12

**Authors:** Wonmin Ko, Nayeon Kim, Hwan Lee, Eun-Rhan Woo, Youn-Chul Kim, Hyuncheol Oh, Dong-Sung Lee

**Affiliations:** 1 College of Pharmacy, Chosun University, Gwangju 61452, Korea; rabis815@naver.com (W.K.); rlaskdus1209@naver.com (N.K.); ghksdldi123@hanmail.net (H.L.); wooer@Chosun.ac.kr (E.-R.W.); 2 Institute of Pharmaceutical Research and Development, College of Pharmacy, Wonkwang University, Iksan 54538, Korea; yckim@wku.ac.kr (Y.-C.K.); hoh@wonkwang.ac.kr (H.O.); 3 Hanbang Cardio-Renal Syndrome Research Center, Wonkwang University, Iksan 54538, Korea

**Keywords:** *Cudrania tricuspidata*, inflammation, ICAM-1, NF-κB, HaCaT

## Abstract

The root bark of *Cudrania tricuspidata* has been reported to have anti-sclerotic, anti-inflammatory, antioxidant, neuroprotective, hepatoprotective, and cytotoxic activities. In the present study, the effect of 16 compounds from *C. tricuspidata* on tumor necrosis factor-α+interferon-γ-treated HaCaT cells were investigated. Among these 16 compounds, 11 decreased IL-6 production and 15 decreased IL-8 production. The six most effective compounds, namely, steppogenin (**2**), cudraflavone C (**6**), macluraxanthone B (**12**), 1,6,7-trihydroxy-2-(1,1-dimethyl-2-propenyl)-3- methoxyxanthone (**13**), cudraflavanone B (**4**), and cudratricusxanthone L (**14**), were selected for further experiments. These six compounds decreased the expression levels of chemokines, such as regulated on activation, normal T cell expressed and secreted (RANTES) and thymus and activation-regulated chemokine (TARC), and downregulated the protein expression levels of intercellular adhesion molecule-1. Compounds **2**, **6**, **12**, **4**, and **14** inhibited nuclear factor-kappa B p65 translocation to the nucleus; however, compound **13** showed no significant effects. In addition, extracellular signal regulatory kinase-1/2 phosphorylation was only inhibited by compound **14**, whereas p38 phosphorylation was inhibited by compounds **13** and **4**. Taken together, the compounds from *C. tricuspidata* showed potential to be further developed as therapeutic agents to suppress inflammation in skin cells.

## 1. Introduction

*Cudrania tricuspidata* Bureau (Moraceae) is a tree that is found in China, Korea, and Japan. Its root has been used in traditional medicines in Korea and China [1]. *C. tricuspidata* is a red apricot that blooms in June and ripens between September and October [2]. Approximately 10 species of *Cudrania* have been identified. These species grow in the wild throughout Korea. The bark of *C. tricuspidata* is grayish brown, its deformed branches have thorns whose length ranges from 0.5 to 3.5 cm, and its trunk has hairs. Its old bark has a yellowish-gray color and is torn vertically [3], and its leaves are split in three. Furthermore, the edges of the leaves are flat and egg-shaped, and both shapes are found on a single tree [4]. *C. tricuspidata* can be used throughout the year. In fact, its roots and leaves contain active pharmaceutical substances that exhibit anticancer, antioxidant, and hypoglycemic effects [5]. The root bark of *C. tricuspidata* has also been reported to exhibit anti-sclerotic [6], neuroprotective [7], anti-inflammatory [8], mast cell activation [9], cytotoxic [10], pancreatic lipase inhibitory [11], and monoamine oxidase suppressive effects [12]. Several parts of *C. tricuspidata* have been studied regarding their phytochemical characterization, standing out its abundancy on phenolic compounds, especially flavonoids. In this regard, flavanone, flavonol, flavonol glycoside, flavanone glycoside, and xanthone were already reported by different studies highlighting the antioxidant potential of the phenolic-rich extracts obtained from *C. tricuspidata* samples [13,14,15,16,17,18,19,20,21]. Keratinocytes are known to play an important role in inflammation [22]. Keratinocytes secrete various cytokines and chemokines, causing a local inflammatory reaction [23]. In fact, many skin diseases, such as allergic contact dermatitis, psoriasis, and atopic dermatitis, are related to inflammation. Dermatitis is affected by genetic, environmental, and immunological disorders, while chronic inflammation is characterized by increased epidermal thickness and the infiltration of macrophages, mast cells, and other inflammatory cells [24]. The stimulation of keratinocytes by tumor necrosis factor α (TNF-α) and interferon γ (IFN-γ) is highly dependent on their activation and this stimulation induces the expression and secretion of chemokines, such as regulated on activation, normal T cell expressed and secreted (RANTES) and thymus and activation-regulated chemokine (TARC), which are regulated accordingly. These factors contribute to the recruitment and infiltration of inflammatory cells in the skin [25]. The stimulation of keratinocytes by TNF-α and IFN-γ induces the expression of pro-inflammatory cytokines, such as IL-6 and IL-8. These cytokines and chemokines contribute to the infiltration of inflammatory cells into inflamed areas of the skin [26]. Mitogen-activated protein kinases (MAPKs) are important enzymes in cell signaling, apoptosis, carcinogenesis, and the pathogenesis of different diseases [27]. MAPK is a serine/threonine kinase that comprises the following subfamilies: extracellular signal regulatory kinase (ERK), p38 kinase, and c-Jun N-terminal kinase (JNK) [28]. MAPK activation can cause the translocation of c-Fos or c-Jun to the nucleus, where it activates transcription factors via phosphorylation, ultimately causing gene expression changes [28]. Nuclear factor-kappa B (NF-κB) is one of the most important transcription factors that translocates to the nucleus upon activation and causes genetic modifications [29]. After NF-κB translocates to the nucleus, it binds to its DNA recognition element and activates the transcription of its target gene [30]. NF-κB participates in the regulation of various biological processes, such as immune response, cell growth, and apoptosis. The five well-known mammalian NF-κB/Rel proteins are Rel (c-Rel), p65 (RelA), RelB, p50 (NFκB1), and p52 (NFκB2) [30]. NF-κB activation induces a high rate of IκB phosphorylation and proteolysis by IκB kinase (IKK) [30]. Furthermore, at least five distinct IκB proteins, including IκBα, IκBβ, and bcl-3, are known. The purpose of this study was to investigate the effects of 16 compounds from *C. tricuspidata* on skin inflammation.

## 2. Results and Discussion

### 2.1. Chemical Structures of 16 Compounds Isolated from C. tricuspidata

**Figure 1:**
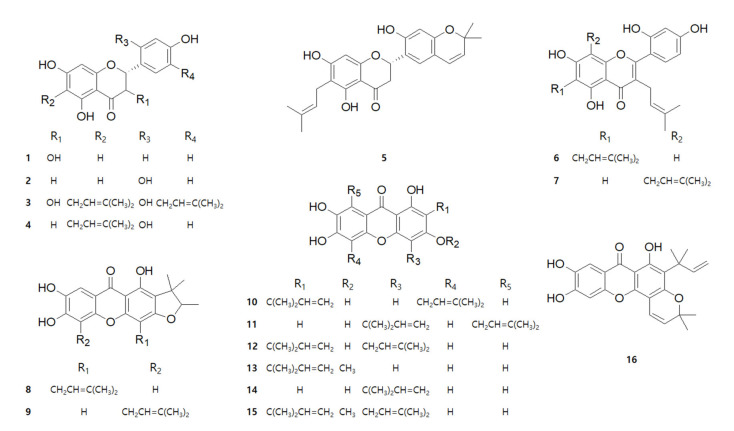
Structure of compounds **1**–**16**.

**Figure 2:**
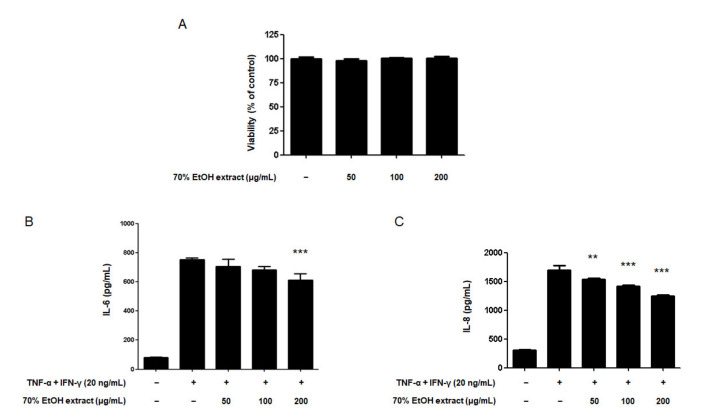
Effects of the 70% EtOH extract from *Cudrania tricuspidata* on the viability of HaCaT cells (**A**) and, IL-6 (**B**) and IL-8 (**C**) production in HaCaT cells stimulated with TNF-α + IFN-γ. (**A**): The cells were incubated with the indicated concentrations for 24 h; (**B**): cells were pre-treated with the 70% EtOH extract for 3 h and stimulated with TNF-α + IFN-γ for 24 h. Each value represents the mean ± SD. ** *p* < 0.01 and *** *p* <0.001 as compared with TNF-α + IFN-γ only.

A total of 16 compounds were isolated from the roots of *C. tricuspidata* in a previous study [31]. The chemical structures and names of the compounds are shown in Figure 1 and Table 1, respectively. *C. tricuspidata* extracts have been reported to have a skin inflammatory modulating effect [3]. Therefore, the inhibitory effects of IL-6 and IL-8 production by the *C. tricuspidata* 70% EtOH extracts were examined (Figure 2). *C. tricuspidata* 70% EtOH extracts had a significant inhibitory effect on IL-6 and IL-8 production against the TNF-α + IFN-γ stimulation in HaCaT cells (Figure 2). Based on these results, it was concluded that there would be some compounds that had an inhibitory effect on skin inflammatory action among the compounds from *C. tricuspidata*.

### 2.2. Effect of the 16 Compounds from C. tricuspidata on the Viability of HaCaT Cells

The cytotoxicity of the 16 compounds was evaluated using a Cell Counting Kit (CCK)-8 assay on HaCaT cells. This assay is a sensitive colorimetric assay for the determination of cell viability in cell proliferation and cytotoxicity assays [32]. Initially, the cell toxicity of all compounds was evaluated at various concentration ranges (1~80 µM; data not shown). The concentration values of all the tested compounds that did not result in cytotoxicity were determined, and then the cell viability was rechecked (Table 2). Therefore, these concentration values were used in subsequent experiments.

### 2.3. Effects of the 16 Compounds from C. tricuspidata on IL-6 Production in TNF-α + IFN-γ-Treated HaCaT Cells

The skin consists of two basic layers: the epidermis and dermis. The epidermis is mainly composed of keratinocytes [33]. Pro-inflammatory cytokines are known to play an important role in the immune response in keratinocytes [16]. Previous studies have reported the roles of TNF-α and IFN-γ in inducing NF-κB activation, which upregulates the production of IL-6 and IL-8 [25]. Initially, it was confirmed that the treatment with each of the compounds alone did not affect IL-6 production (Figure S1). Accordingly, the effect of the 16 compounds from *C. tricuspidata* on IL-6 production in TNF-α + IFN-γ-treated HaCaT cells were determined. Briefly, HaCaT cells were pre-incubated with different concentrations of the 16 compounds for 3 h, and then stimulated with TNF-α + IFN-γ for 24 h. The enzyme immunoassay results indicate that compounds **1**, **2**, **3**, **4**, **6**, **7**, **10**, **12**, **13**, **14**, and **15** significantly decreased IL-6 production (Table 3, Figure S2). This suggested that some compounds isolated from *C. tricuspidata* could be a candidate material for inhibiting skin inflammation.

### 2.4. Effects of the 16 Compounds from C. tricuspidata on IL-8 Production in TNF-α + IFN-γ-Treated HaCaT Cells

Next, the effect of the 16 compounds from *C. tricuspidata* on IL-8 production in HaCaT cells were determined. Briefly, HaCaT cells were pre-incubated with different concentrations of the 16 compounds for 3 h. Thereafter, the cells were subjected to TNF-α + IFN-γ stimulation for 24 h. The enzyme immunoassay revealed that 15 compounds from *C. tricuspidata*, namely, compounds **1**, **2**, **3**, **4**, **5**, **6**, **7**, **8**, **9**, **10**, **12**, **13**, **14**, **15** and **16**, decreased IL-8 production in HaCaT cells (Table 3, Figure S3). Among the 16 compounds, **2**, **6**, **12**, **13**, **4** and **14** showed the most remarkable effect in inhibiting the production of both IL-6 and IL-8 in HaCaT cells (Footnote b in Table 3). Therefore, the most effective six compounds were selected for further experiments. Compound **2**, with its flavanone skeletons, has been studied for its cyclooxygenase [33] and tyrosinase inhibitory effects [34,35,36,37,38]. In a previous study, compound **2** altered the anti-neuroinflammatory response in microglia [39]. Compound **6**, a prenylated flavanone, has been reported to have apoptotic effects on A375.S2 melanoma cells [40] and to lead to the induction of apoptotic cell death in KM12, Caco-2, HT29, HCC2998, HCT116, and SW48 cell lines [41]. Compound **12**, which is a prenylated xanthone, has been shown to exert anti-human immunodeficiency virus (HIV) [42], hepatoprotective [43], cytotoxic [44], and antitumor effects [45]. Compounds **13** and **14**, which are prenylated xanthones, have only been shown to exert an NO production inhibitory effect in BV2 microglia [1]. In a previous study, compound **4**, a prenylated flavanone, has exhibited anti-inflammatory and anti-neuroinflammatory effects in RAW264.7 and BV2 cells [46]. This is the first report on the biological action of compounds **2**, **6**, **12**, **13**, **4**, and **14** on the inhibitory effects of skin inflammatory factors in skin HaCaT cells. Therefore, further mechanism studies of these six compounds in HaCaT cells were conducted.

### 2.5. Effects of the Six Compounds from C. tricuspidata on RANTES and TARC Production by TNF-α + IFN-γ-Treated HaCaT Cells

**Figure 3:**
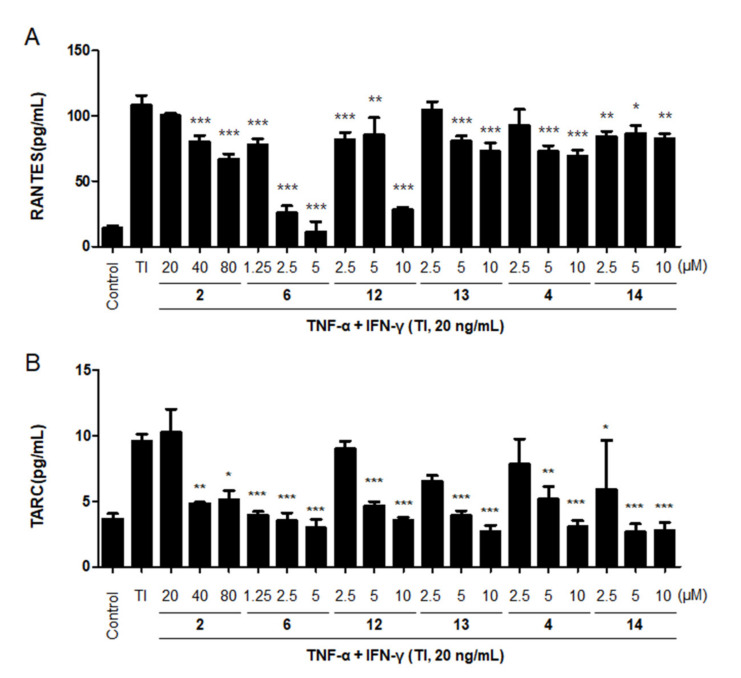
Effects of the six compounds from *C. tricuspidata* on RANTES (**A**) and TARC (**B**) production in TNF-α + IFN-γ-treated HaCaT cells. HaCaT cells were incubated with TNF-α + IFN-γ (20 ng/mL) in the presence or absence of the six compounds at the indicated concentrations. After 24 h, the chemokine secretion levels were analyzed using enzyme-linked immunosorbent assay (ELISA). Data are presented as the mean ± standard deviation. * *p* < 0.05, ** *p* < 0.01, and *** *p* < 0.001 compared with TNF-α + IFN-γ only. TI: TNF-α + IFN-γ.

Among the 16 compounds, the six most effective compounds were selected for further experiments on the production of the representative Th2 chemokines, RANTES (CCL5) and TARC (CCL17). The transport of Th2 cells to inflamed sites via CC chemokine receptor 4 (CCR4) is known to induce cell migration and invasion [47]. In many well-known skin immune diseases, an increase in the cytokine and chemokine levels is known as a biomarker of chronic inflammation; therefore, the suppression of these cytokines and chemokines may play a critical role in treating inflammatory skin diseases [48,49,50]. TNF-α and IFN-γ have a major role in inducing NF-κB and STAT1 activation, which upregulates the expression of various chemokines, including MDC/CCL22, TARC/CCL17, RANTES/CCL5, and IL-8 [51]. These chemokines or cytokines were elevated in the skin of patients and suggested as biomarkers for risk stratification of skin inflammation [52]. HaCaT cells were pre-incubated with different concentrations of the six compounds from *C. tricuspidata* for 3 h. Thereafter, the cells were stimulated with TNF-α + IFN-γ for 24 h. The increased production of RANTES due to TNF-α + IFN-γ treatment was found to be decreased by the pretreatment with all six compounds (Figure 3A). Similarly, TARC production induced by TNF-α + IFN-γ (20 ng/mL) was decreased by the pretreatment with all six compounds from *C. tricuspidata* (Figure 3B). Based on this result, it is considered that six compounds from *C. tricuspidata* have excellent inhibitory effects on the production of chemokines and cytokines. Therefore, by focusing on this point, further mechanism experiments were conducted using by six compounds.

### 2.6. Effects of the Six Compounds from C. tricuspidata on COX-2 and ICAM-1 Protein Production by TNF-α + IFN-γ-Treated HaCaT Cells

**Figure 4:**
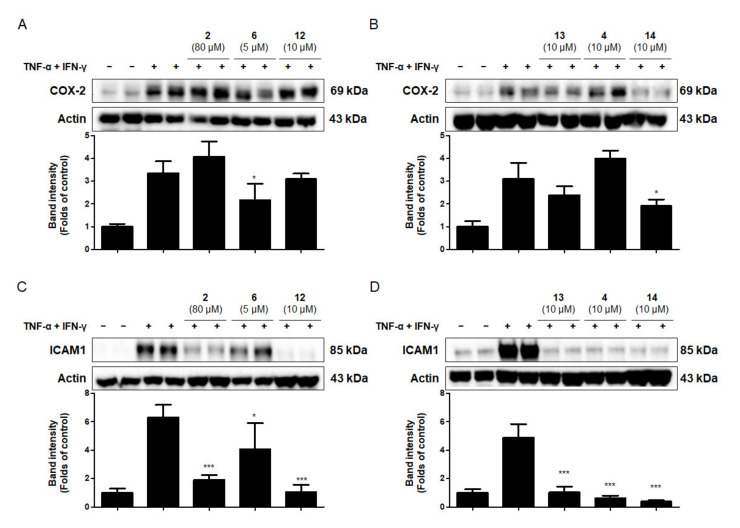
Protein expression levels of COX-2 (**A**,**B**) and ICAM-1 (**C**,**D**) in TNF-α + IFN-γ-treated HaCaT cells. (**A**,**B**) Cells were pretreated with the six compounds from *C. tricuspidata* at the indicated concentrations for 3 h and stimulated with TNF-α + IFN-γ (20 ng/mL) for 24 h. (**C**,**D**) Cells were pretreated with the six compounds from *C. tricuspidata* at the indicated concentrations for 3 h, and then stimulated with TNF-α + IFN-γ (20 ng/mL) for 6 h. Western blotting analysis was performed as described in the Materials and Methods section. Representative stains from four independent experiments are presented. The immunoblot was quantified using ImageJ software. The band intensity was normalized to that of actin. * *p* < 0.05, *** *p* < 0.001 compared with TNF-α + IFN-γ only. COX-2, cyclooxygenase-2; ICAM-1, intercellular adhesion molecule-1.

Intercellular adhesion molecule (ICAM)-1 is upregulated in response to inflammatory mediators, such as the pro-inflammatory cytokines IL-1β, TNF-α, and IFN-γ [36]. ICAM-1 is expressed in keratinocytes and is critical for the development of many skin diseases, such as contact dermatitis and lichen planus [53]. Cyclooxygenase (COX) is the enzyme responsible for the conversion of arachidonic acid to prostaglandin H2, which is the main step in the prostaglandin synthesis pathway. COX-2 expression plays a key role in skin inflammation [54]. In this experiment, the anti-inflammatory effects of the six compounds from *C. tricuspidata* on the expression of pro-inflammatory proteins were investigated in TNF-α + IFN-γ-treated HaCaT cells. Briefly, cells were pre-incubated for 3 h with or without the six compounds from *C. tricuspidata* (5–80 μM) and were subsequently challenged with TNF-α + IFN-γ (20 ng/mL) for 24 h. As shown in Figure 4A,B, compounds **6** and **14** decreased the expression level of COX-2. Previous studies have reported that compound **6** inhibits the expression of COX-2 [55]; however, this study is the first to report the inhibition of COX-2 protein production by compound **6** in HaCaT cells. In addition, cells were pre-incubated for 3 h with or without the six compounds from *C. tricuspidata* (5–80 μM), and subsequently challenged with TNF-α + IFN-γ (20 ng/mL) for 6 h. All six compounds significantly inhibited ICAM-1 expression in TNF-α + IFN-γ-treated HaCaT cells (Figure 4C,D). Among the six compounds, compounds **6** and **14** were particularly effective in inhibiting COX-2 and ICAM-1 expression. These results suggest that compounds **6** and **14** could inhibit chemokines and cytokines by inhibiting COX-2 and ICAM-1 expression in skin cells.

### 2.7. Effects of the Six Compounds from C. tricuspidata on NF-κB Activation in TNF-α + IFN-γ-Treated HaCaT Cells

**Figure 5:**
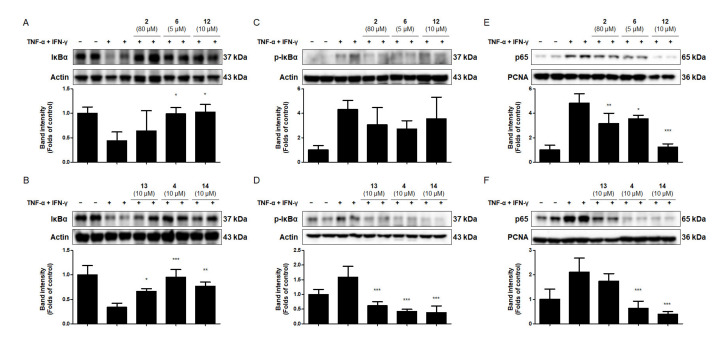
Effects of the six compounds from *C. tricuspidata* on the degradation of IκB-α (**A**,**B**), the phosphorylation of IκBα (**C**,**D**), and the translocation of NF-κB p65 to the nucleus (**E**,**F**) in HaCaT cells. Cells were pretreated with the six compounds from *C. tricuspidata* at the indicated concentrations for 3 h and stimulated with TNF-α + IFN-γ (20 ng/mL) for 10 min. IκBα, p-IκBα, and NF-κB p65 were analyzed using Western blotting, as described in the Materials and Methods section. Representative blots from four independent experiments are presented. The immunoblots were quantified using ImageJ software. The band intensity was normalized to that of actin or anti-proliferating cell nuclear antigen (PCNA). * *p* < 0.05, ** *p* < 0.01, *** *p* < 0.001 compared with TNF-α + IFN-γ only.

The activation of NF-κB by TNF-α and/or IFN-γ results from high levels of IκB phosphorylation and the degradation of IκB in keratinocytes [29]. NF-κB–mediated inflammation is a significant component of innate immunity and seems to be a final common pathway for the aggravation of the inflammatory response to stimuli in immune diseases of the skin [56]. To determine the potential mechanisms of the inhibition of TNF-α + IFN-γ-induced pro-inflammatory enzyme and the mediator levels by the six compounds from *C. tricuspidata*, their effects on the phosphorylation and degradation of cytoplasmic IκBα and on p65 translocation to the nucleus were investigated. Compounds **6**, **12**, **13**, **4** and **14** were found to inhibit the degradation of IκBα in the cytoplasm (Figure 5A,B). In addition, compounds **13**, **4**, and **14** inhibited the phosphorylation of IκBα (Figure 5C,D). When HaCaT cells were exposed to TNF-α + IFN-γ, NF-κB p65 translocated from the cytoplasm to the nucleus. Among the six compounds tested, five compounds (**2**, **6**, **12**, **4** and **14**) significantly inhibited NF-κB p65 translocation to the nucleus; however, compound **13** showed no significant effect (Figure 5E,F). These findings suggest that compounds **2**, **6**, **12**, **13**, **4** and **14** were useful in preventing skin inflammation, acting as NF-κB inhibitors. In particular, compounds **6** and **14** inhibited COX-2 and ICAM-1 expression, and also significantly inhibited NF-κB p65 translocation. Through this, these results suggest that compounds **6** and **14** have significantly affected in regulating of upstream signaling factors in skin inflammation.

### 2.8. Effects of the Six Compounds from C. tricuspidata on MAPK Phosphorylation in TNF-α + IFN-γ-Treated HaCaT Cells

**Figure 6:**
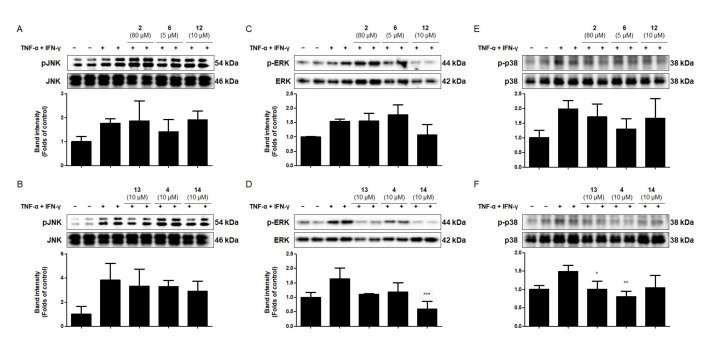
Effect of the six compounds from *C. tricuspidata* on JNK-1/2, ERK-1/2, and p38 phosphorylation in HaCaT cells. Cells were pretreated with the six compounds at the indicated concentrations for 3 h, and then stimulated with TNF-α + IFN-γ (20 ng/mL) for 2 h (**A**,**B**), 15 min (**C**,**D**), or 6 h (**E**,**F**). Cell extracts were analyzed using Western blotting and antibodies specific for phosphorylated JNK1/2 (p-JNK1/2), p-ERK1/2, or p-p38. The membrane was stripped and reprobed to determine the total abundance of each MAPK, as a control measure. Representative blots from four independent experiments are presented. Immunoblots were quantified using ImageJ software. The band intensity was quantified and normalized to each total protein concentration. * *p* < 0.05, ** *p* < 0.01, and *** *p* < 0.001 compared with TNF-α + IFN-γ only. JNK, c-Jun N-terminal kinase; ERK, extracellular signal regulatory kinase; MAPK, mitogen-activated protein kinase.

Stimulation of keratinocytes with TNF-α + IFN-γ upregulates various MAPK signaling pathways [57,58]. To evaluate the anti-inflammatory effects of the six compounds from *C. tricuspidata* on the MAPK pathway, Western blotting analysis was used to determine the phosphorylation levels of JNK1/2, ERK1/2, and p38 in TNF-α + IFN-γ-treated HaCaT cells. Cells were pretreated with the six compounds from *C. tricuspidata* at the indicated concentrations for 3 h, and then stimulated with TNF-α + IFN-γ (20 ng/mL) for either 15 min, 2 h, or 6 h. Following the treatment with TNF-α + IFN-γ for 2 h, the phosphorylation of JNK1/2 was not inhibited by any of the six compounds (Figure 6A,B). After 15 min of treatment with TNF-α + IFN-γ, ERK1/2 phosphorylation was inhibited only by compound **14** (Figure 6C,D). The phosphorylation of p38 was inhibited only by compounds **13** and **4** after 6 h of treatment with TNF-α + IFN-γ (Figure 6E,F). The potential of MAPKs activation to negatively regulate proinflammatory mediators has also been reported in some previous studies, in which ERK1/2 or p38 has been implicated as an anti-inflammatory signal [59,60]. Therefore, this study suggests that compound **14**, **13** and **4** mediated ERK1/2 or p38 phosphorylation could serve as an important mechanism by which the compounds exerts its anti-inflammatory effects in skin cells. In addition, these results indicate that the compounds from *C. tricuspidata* inhibited TNF-α + IFN-γ-induced NF-κB and MAPK activation, as well as the production of cytokines and chemokines.

## 3. Materials and Methods

### 3.1. Chemicals and Reagents for Cell Culture

Phosphate-buffered saline (PBS), fetal bovine serum (FBS), penicillin, streptomycin, and DMEM + GlutaMAX^TM^, which were used for cell culture, were purchased from Gibco (Grand Island, NY, USA). All other chemicals were purchased from Sigma-Aldrich (St. Louis, MO, USA). Primary antibodies, including anti-COX-2, anti-ICAM-1, anti-IκBα, anti-p-IκBα, anti-p65, anti-actin, and anti-proliferating cell nuclear antigen, were purchased from Santa Cruz Biotechnology (Dallas, TX, USA), and anti-p-JNK, anti-JNK, anti-p-ERK, anti-ERK, anti-p-p38, and anti-p38 were purchased from Cell Signaling Technology (Danvers, MA, USA). Anti-rabbit and anti-mouse horseradish peroxidase-conjugated secondary antibodies were purchased from Millipore (Billerica, MA, USA). The isolation and structural determination of the 16 compounds from *C. tricuspidata* have been described elsewhere [31].

### 3.2. Cell Culture

HaCaT cells were donated by Hyeonsook Cheong, Chosun University (Kwangju, Korea). Cells (5 × 10^6^ cells/dish) were seeded on 100-mm dishes in DMEM + GlutaMAX^TM^ containing streptomycin (100 μg/mL), 10% heat-inactivated FBS, and penicillin G (100 units/mL), and then incubated at 37 °C in a humidified atmosphere (5% CO_2_ and 95% air). TNF-α + IFN-γ (20 ng/mL) was used as negative control.

### 3.3. CCK8 Assay

To determine the viability of HaCaT cells, these were maintained at 2 × 10^4^ cells/well, treated with the test compounds in the presence or absence of glutamate (5 mM), and cultured for 24 h. Subsequently, a CCK8 assay (Dojindo Laboratories, Kumamoto, Japan) was performed and the cells were incubated for 1 h. The absorbance was then measured at 450 nm.

### 3.4. Enzyme-Linked Immunosorbent Assay (ELISA)

The cytokines and chemokines in IL-6, IL-8, CCL17 (TARC), and CCL5 (RANTES) HaCaT cells were detected using an enzyme-linked immunosorbent assay. The levels of these cytokines and chemokines were measured using commercial ELISA kits (BioLegend, San Diego, CA, USA), according to the manufacturer’s instructions.

### 3.5. Western Blotting Analysis

Cells were harvested and pelleted via centrifugation at 200× *g* for 3 min, washed with PBS, and lysed in 20 mM Tris-HCl buffer (pH 7.4) containing a protease inhibitor mixture (0.1 mM phenylmethanesulfonyl fluoride, 5 mg/mL aprotinin, 5 mg/mL pepstatin A, and 1 mg/mL chymostatin). The protein concentration was determined using a protein assay dye reagent concentrate (#5000006; Bio-Rad Laboratories, Hercules, CA, USA). A total of 30 micrograms of protein from each sample was resolved using 7.5% and 12% sodium dodecyl sulfate-polyacrylamide gel electrophoresis. Thereafter, the proteins were electrophoretically transferred onto Hybond enhanced chemiluminescence (ECL) nitrocellulose membranes (Bio-Rad Laboratories). The membranes were blocked with 5% skimmed milk, and sequentially incubated with the relevant primary antibody and horseradish peroxidase-conjugated secondary antibody, prior to ECL detection (Pierce Biotechnology, Rockford, IL, USA).

### 3.6. Preparation of the Cytosolic and Nuclear Fractions

HaCaT cells were homogenized in PER-Mammalian Protein Extraction Buffer (1:20, w:v) (Pierce Biotechnology, Rockford, IL, USA) containing freshly added protease inhibitor cocktail I (EMD Biosciences, San Diego, CA, USA) and 1 mM phenylmethanesulfonyl fluoride. The cytosolic fractions were prepared using centrifugation at 16,000× *g* for 5 min at 4 °C, while the nuclear and cytoplasmic cell extracts were prepared using NE-PER nuclear and cytoplasmic extraction reagents, respectively (Pierce Biotechnology, Rockford, IL, USA).

### 3.7. Statistical Analysis

Data are expressed as the mean ± standard deviation of three independent experiments. Statistical analysis was performed using GraphPad Prism software (version 5.01; GraphPad Software Inc., San Diego, CA, USA). The differences between means were assessed using one-way analysis of variance, followed by Tukey’s multiple comparison test. A *p* value < 0.05 was used to indicate statistical significance.

## 4. Conclusions

In this study, the effects of 16 compounds from the roots of *C. tricuspidata* on skin inflammation were examined. Among the 16 compounds tested to decrease IL-6 and IL-8 production, the most effective six compounds, namely, steppogenin (**2**), cudraflavone C (**6**), macluraxanthone B (**12**), 1,6,7-trihydroxy-2-(1,1-dimethyl-2-propenyl)-3-methoxy xanthone (**13**), cudraflavanone B (**4**), and cudratricusxanthone L (**14**), were selected for subsequent experiments, and TNF-α + IFN-γ-induced RANTES, TARC, and ICAM-1 expression levels were decreased by the six compounds. However, only compounds **6** and **14** were found to reduce the expression levels of COX-2. Furthermore, the compounds **2**, **6**, **12**, **4**, and **14** significantly inhibited NF-κB p65 translocation to the nucleus. These results demonstrate that the compounds from *C. tricuspidata* could be further developed as therapeutic agents that suppress inflammation in human keratinocytes. These data may be used as the basis for the development of effective therapeutic strategies for skin inflammatory diseases.

## Figures and Tables

**Table 1:** List of the 16 compounds evaluated in this study.

Compound	Name
**1**	dihydrokaempferol
**2**	steppogenin
**3**	cudraflavanone D
**4**	cudraflavanone B
**5**	cudraflavanone A
**6**	cudraflavone C
**7**	kuwanon C
**8**	cudraxanthone M
**9**	cudraxanthone D
**10**	cudraxanthone L
**11**	cudratricusxanthone A
**12**	macluraxanthone B
**13**	1,6,7-trihydroxy-2-(1,1-dimethyl-2-propenyl)-3-methoxyxanthone
**14**	cudratricusxanthone L
**15**	cudracuspixanthone A
**16**	cudratricusxanthone N

**Table 2:** Effects of the 16 compounds from *C. tricuspidata* on the viability of HaCaT cells.

Compounds	Name	Cytotoxicity (μM)	
**1**	dihydrokaempferol	>80	>80
**2**	steppogenin	>80	>80
**3**	cudraflavanone D	>10	>10
**4**	cudraflavanone B	>20	>20
**5**	cudraflavanone A	>40	>40
**6**	cudraflavone C	>5	>5
**7**	kuwanon C	>40	>40
**8**	cudraxanthone M	>10	>10
**9**	cudraxanthone D	>5	>5
**10**	cudraxanthone L	>2	>2
**11**	cudratricusxanthone A	>5	>5
**12**	macluraxanthone B	>5	>5
**13**	1,6,7-trihydroxy-2-(1,1-dimethyl-2-propenyl)-3-methoxyxanthone	>40	>40
**14**	cudratricusxanthone L	>20	>20
**15**	cudracuspixanthone A	>2	>2
**16**	cudratricusxanthone N	>2	>2

**Table 3:** Inhibitory effects of the 16 compounds on IL-6 and IL-8 production in TNF-α + IFN-γ-treated HaCaT cells.

Compounds	Name	IC_50_ (μM)	
		IL-6	IL-8
**1**	dihydrokaempferol	>80 ^a^	>80 ^a^
**2** ^b^	steppogenin	>80 ^a^	62.17 ± 16.46 ^a^
**3**	cudraflavanone D	>10 ^a^	4.20 ± 1.03 ^a^
**4** ^b^	cudraflavanone B	>20 ^a^	9.88 ± 3.22 ^a^
**5**	cudraflavanone A	>40	>40 ^a^
**6** ^b^	cudraflavone C	2.78 ± 0.75 ^a^	2.16 ± 0.50 ^a^
**7**	kuwanon C	25.75 ± 5.16 ^a^	>40 ^a^
**8**	cudraxanthone M	>10	>10 ^a^
**9**	cudraxanthone D	>5 ^a^	>5 ^a^
**10**	cudraxanthone L	>2 ^a^	>2 ^a^
**11**	cudratricusxanthone A	>5	>5
**12** ^b^	macluraxanthone B	>5 ^a^	7.88 ± 3.09 ^a^
**13** ^b^	1,6,7-trihydroxy-2-(1,1-dimethyl-2-propenyl)-3-methoxyxanthone	9.69 ± 2.15 ^a^	3.87 ± 0.96 ^a^
**14** ^b^	cudratricusxanthone L	9.86 ± 0.92 ^a^	3.70 ± 0.70 ^a^
**15**	cudracuspixanthone A	>2 ^a^	1.54 ± 0.53 ^a^
**16**	cudratricusxanthone N	>2	>2 ^a^
**Positive control**	curcumin	11.96 ± 3.13	10.29 ± 2.20

^a^ inhibitory effective compounds, *p* < 0.05; ^b^ six selected compounds with the best inhibitory effect; the half maximal inhibitory concentration (IC_50_).

## Data Availability

The data presented in this study are available on request from the corresponding author.

## Supplementary Materials

The following are available online at https://www.mdpi.com/article/10.3390/ijms22147472/s1.
