# Supplementary material for: Anti-Inflammatory Effects of Compounds from Cudrania tricuspidata in HaCaT Human Keratinocytes
Source: Int J Mol Sci. 2021 Jul 12;22(14):7472. doi: 10.3390/ijms22147472 (PMC8303187; doi:10.3390/ijms22147472)
Supplement: Supplementary file 1 [file ijms-22-07472-s001.zip › ijms-1257922-supplementary.pdf]

International Journal of
Molecular Sciences

Article

Anti-Inflammatory Effects of Compounds from *Cudrania tricuspidata* in HaCaT Human Keratinocytes

Wonmin Ko [1,#], Nayeon Kim [1,#], Hwan Lee [1], Eun-Rhan Woo [1], Youn-Chul Kim [2], Hyuncheol Oh [2,3] and Dong-Sung Lee [1,*]

[1] College of Pharmacy, Chosun University, Dong-gu, Gwangju, 61452, Republic of Korea; rabis815@naver.com (W.K.); rlaskdus1209@naver.com (N.K.); ghksdldi123@hanmail.net (H.L.); wooer@Chosun.ac.kr (E.R.W)

[2] Institute of Pharmaceutical Research and Development, College of Pharmacy, Wonkwang University, Iksan 54538, Republic of Korea; yckim@wku.ac.kr (Y.C.K.); hoh@wonkwang.ac.kr (H.O.)

[3] Hanbang Cardio-Renal Syndrome Research Center, Wonkwang University, Iksan 54538, Republic of Korea

* Correspondence: dslee2771@chosun.ac.kr; Tel.: +82-62-230-6386, Fax: +82-62-222-5414

\# These authors contributed equally to this work.

Received: date; Accepted: date; Published: date

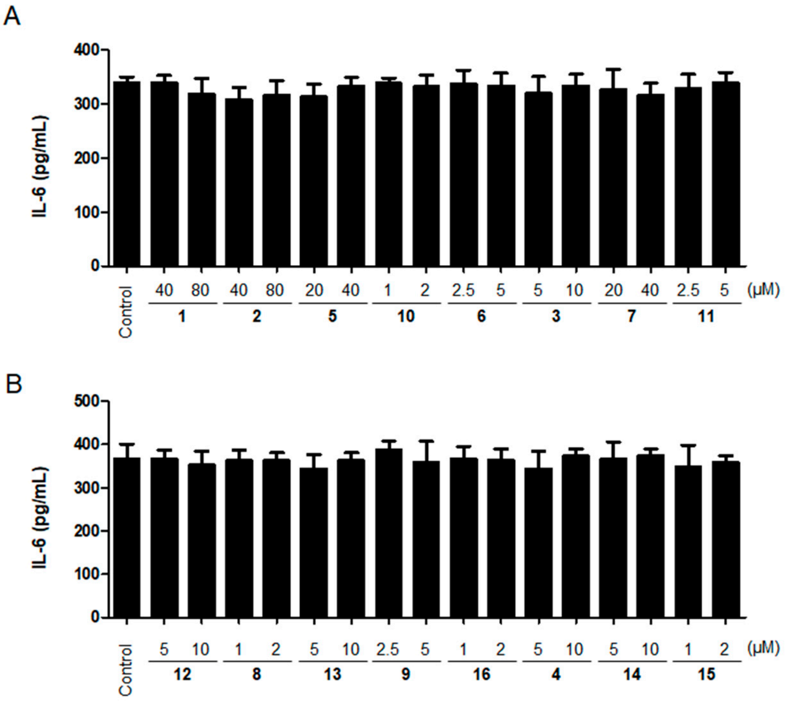

Figure S1. Effects of the 16 compounds from *C. tricuspidata* on IL-6 production in HaCaT cells. HaCaT cells were incubated with the indicated concentrations of the 16 compounds from *C. tricuspidata* only for 24 h. IL-6 levels were determined as described in the Materials and Methods section. The data represent the mean values ± SD of three experiments.

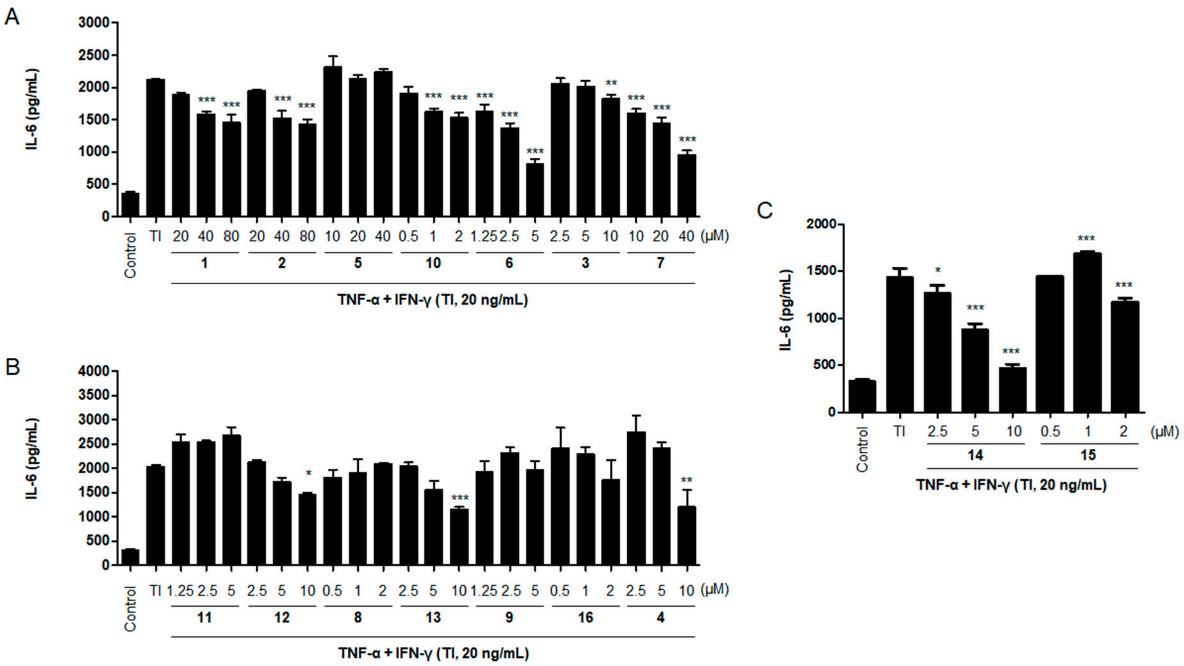

Figure S2. Effects of the 16 compounds from *C. tricuspidata* on IL-6 production in TNF-α+IFN-γ-treated HaCaT cells (A, B, and C). Cells were pre-treated with the indicated concentrations of the 16 compounds from *C. tricuspidata* for 3 h and then stimulated with TNF-α+IFN-γ (20 ng/mL) for 24 h. IL-6 levels were determined as described in the Materials and Methods section. The results are presented as the mean ± standard deviation of three experiments. *$p < 0.05$, **$p < 0.01$, and ***$p < 0.001$ compared with TNF-α+IFN-γ only.

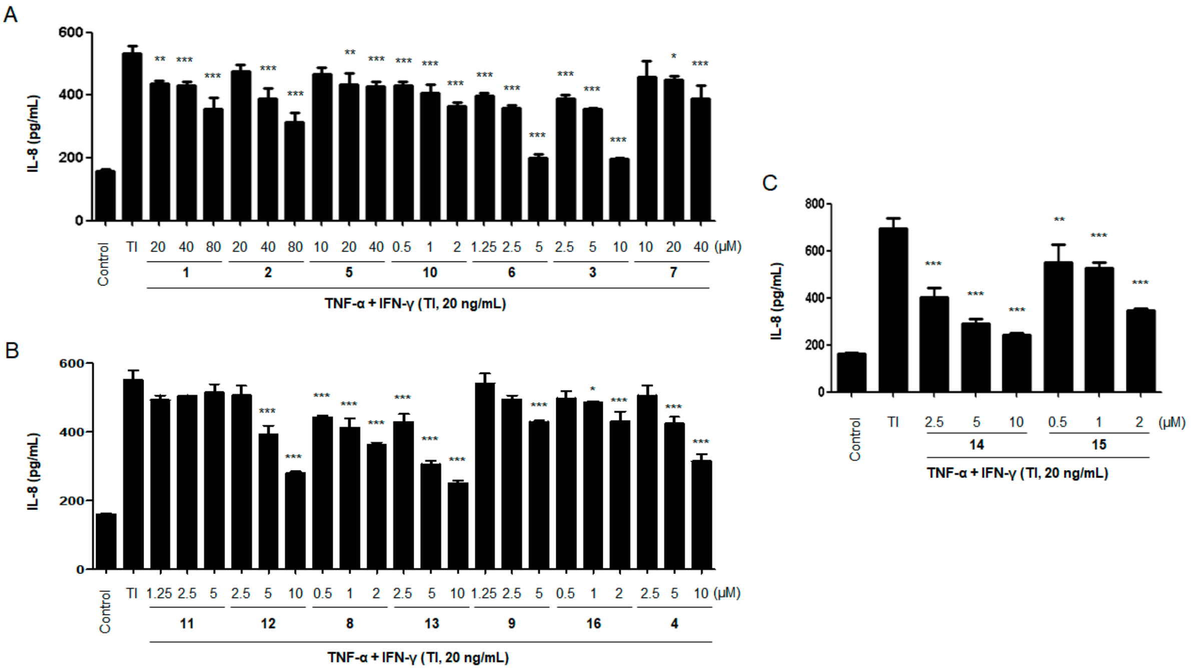

Figure S3. Effects of the 16 compounds from *C. tricuspidata* on IL-8 (A, B, and C) production in TNF-α+IFN-γ-treated HaCaT cells. The cells were pre-treated with the indicated concentrations of the *C. tricuspidata* compounds for 3 h, and then stimulated with TNF-α+IFN-γ (20 ng/mL) for 24 h. IL-6 levels were determined as described in the Materials and Methods section. Data are expressed as the mean ± standard deviation of three experiments. **p* < 0.05, ***p* < 0.01, and ****p* < 0.001 compared with TNF-α+IFN-γ only.
